# Pro-Apoptotic Activity of Bioactive Compounds from Seaweeds: Promising Sources for Developing Novel Anticancer Drugs

**DOI:** 10.3390/md21030182

**Published:** 2023-03-15

**Authors:** Rosette Agena, Alejandro de Jesús Cortés-Sánchez, Humberto Hernández-Sánchez, María Eugenia Jaramillo-Flores

**Affiliations:** 1 Ingeniería Bioquímica, Escuela Nacional de Ciencias Biológicas (ENCB), Instituto Politécnico Nacional, Mexico City 07738, Mexico; 2 Consejo Nacional de Ciencia y Tecnología (CONACYT), Universidad Autónoma Metropolitana, Unidad Lerma (UAMLerma), Municipio Lerma de Villada C.P. 52005, Mexico

**Keywords:** antioxidant activity, apoptosis, Bcl-2 family proteins, bioactive compounds, cancer, caspases, extrinsic pathway, intrinsic pathway, ROS, seaweeds

## Abstract

The process by which cancer cells evade or inhibit apoptosis is considered one of the characteristics of cancer. The ability of cancer cells to escape apoptosis contributes to tumor proliferation and promotes metastasis. The discovery of new antitumor agents is essential for cancer treatment due to the lack of selectivity of drugs and cellular resistance to anticancer agents. Several studies showed that macroalgae produce various metabolites with different biological activities among marine organisms. This review discusses multiple metabolites extracted from macroalgae and their pro-apoptotic effects through regulating apoptosis signaling pathway target molecules and the structure-activity relationship. Twenty-four promising bioactive compounds have been reported, where eight of these compounds exhibited values of maximum inhibitory concentration (IC50) of less than 7 μg/mL. Fucoxanthin was the only carotenoid reported that induced apoptosis in HeLa cells with an IC50 below 1 µg/mL. Se-PPC (a complex of proteins and selenylated polysaccharides) is the magistral compound because it is the only one with an IC50 of 2.5 µg/mL which regulates the primary proteins and critical genes of both apoptosis pathways. Therefore, this review will help provide the basis for further studies and the development of new anticancer drugs, both as single agents and adjuvants, decreasing the aggressiveness of first-line drugs and offering patients better survival and quality of life.

## 1. Introduction

Cancer is one of the diseases with the highest mortality and morbidity rates worldwide. In developed countries, it is the second leading cause of death after cardiovascular disease and the third in developing countries after cardiovascular diseases and parasitic infections. By 2040, it is estimated that cancer incidence will grow to more than 28.4 million, with an increase of 47% compared to 2020 [1].

Cancer is a disease characterized by the uncontrolled and continuous growth of cells that form tumors with the ability to generate metastases. Moreover, cancer leads to an imbalance between cell division and death. The cell does not respond to the apoptotic signals and acquires inherent properties. Under this circumstance, the cell evades the intrinsic and extrinsic mechanisms that control carcinogenesis. Continuous and excessive cell proliferation and death avoidance are characteristics of cancer that actively and directly participate in the transformation of a tumor cell by providing various mechanisms that drive cancer progression that could be used as a vehicle for targeted cancer treatment [2]. Therefore, searching for new natural compounds with anti-cancer activity is urgent [3]. Natural compounds have been shown to have a better affinity to interact with biological systems, lower development costs, and reduced potential side effects [4,5].

It has been reported that approximately 60% of cancer drugs are derived from plants, bacteria, and marine organisms [5]. Macroalgae are the base of the food chain, photosynthetic, with a cosmopolitan distribution. They are divided into three large groups according to their coloration: *Chlorophyta* (green algae), which totals between 6000 and 7000 species; *Rhodophyta* (red algae), with more than 3000 species; and *Ochrophyta* (brown or brown algae), which has around 2000 species and are giant algae [6]. Macroalgae have been used in cooking and traditional medicine in East Asian countries where a lower incidence of chronic diseases, such as hyperlipidemia, coronary heart disease, diabetes, and cancer, has been observed compared to Western countries [7]. In addition, compared to plants, they have the advantages of faster cultivation, processing, and harvesting cycle. They can be grown in waste materials, improving drug profitability and biological potential [8]. They are a vital source of nutrients such as fatty acids, carbohydrates, minerals, proteins, and vitamins [9]. On the other hand, they produce various secondary metabolites such as alkaloids, carotenoids, phenolic compounds, steroids, phytosterols, glycoproteins, and terpenoids, among others, in response to ecological competition and adverse environmental conditions [10]. These compounds have been shown to have various biological activities: antiangiogenic, anti-inflammatory, antimutagenic, antioxidant, antiproliferative, antitumor, antifungal, anticoagulant, antiviral, and antihypertensive, among others [11]. The beneficial effects of compounds extracted from macroalgae on cancer have been increasingly explored, making them an alternative for designing new drugs and/or adjuvants of first-line drugs, decreasing their aggressiveness. Therefore, this review summarizes and discusses the pro-apoptotic activity of different compounds extracted from macroalgae on cancer cell lines and the relationship between these compounds’ chemical structure and pro-apoptotic and antioxidant activity.

## 2. Apoptosis: A Target for Anticancer Therapy

Apoptosis is a regulated cell death process executed by two alternative pathways: extrinsic and intrinsic. However, evading death by different mechanisms is an essential molecular aspect of carcinogenesis. Therefore, inducing apoptosis became one of the essential objectives of cancer therapy. Death receptors, adaptor proteins, FLIP, NF-κB, pro- and anti-apoptotic proteins of the B cell lymphoma (Bcl)-2 family, intracellular ROS and Ca^2+^ levels, mitochondrial membrane permeability, p53, and initiating and effector caspases are considered the most critical targets of both apoptosis pathways.

### 2.1. The Extrinsic or Death Receptor Pathway

The extrinsic pathway is triggered when the death ligands, including FasL, tumor necrosis factor (TNF), and TNF-related apoptosis-inducing ligand (TRAIL), bind to their corresponding death receptors Fas, tumor necrosis factor receptor (TNFR), and TNF-related apoptosis-inducing ligand receptor 1/2 (TRAIL-R1/2 or death receptor 4/5 (DR4/5). The intracellular death domains of these complexes engage adaptor proteins such as Fas-associated protein with death domain (FADD) and TNFR1-associated death domain protein (TRADD), as well as caspase-8 or -10, forming the death-inducing signaling complex (DISC). This complex activates the apoptosis-initiating caspases -8 and -10, activating executioner caspases-3, -6, and -7 [12]. Caspases are a group of enzymes of the cysteine protease family capable of hydrolyzing tetrapeptides containing an aspartic acid residue. They are synthesized as zymogens (inactive proenzymes or pro-caspases) that are converted into the active form by proteolytic breakage, acquiring their catalytic capacity to degrade their target substrates. The initial activation of a caspase causes a chain reaction that leads to the activation of other caspases and cell death [13].

**Figure 1:**
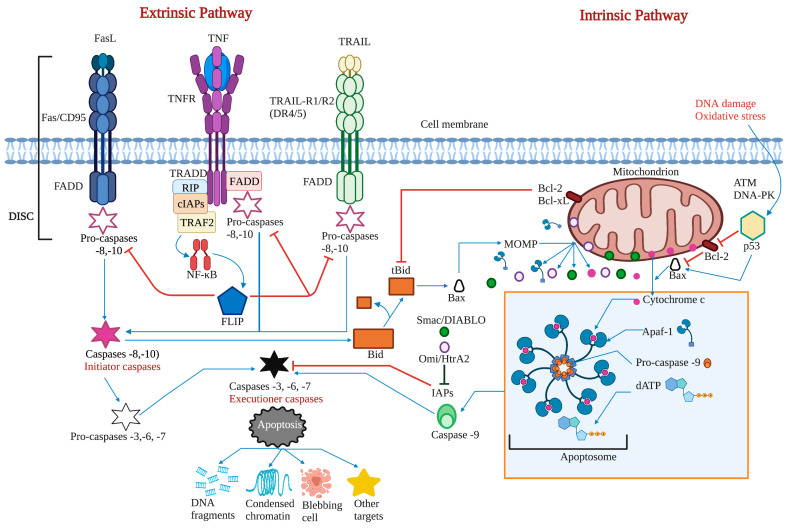
The extrinsic and intrinsic pathways of apoptosis. Taken and modified from D’Arcy [12]. The extrinsic pathway is triggered by external stimuli or ligand molecules, particularly involving death receptors. The intrinsic pathway is mediated by Bax/Bak pore formation into the mitochondrial membrane. Subsequently, cytochrome *c* is released and combines with apoptotic protease activating factor-1 (Apaf)–1 and procaspase-9 to form the apoptosome. Both pathways converge in activating executioner caspases -3, -6, and -7.

While the TNF pathway leads to apoptosis, it can also activate a nuclear transcription factor-dependent signaling pathway (NF-κB), leading to cell survival. NF-κB is inactive in the cytoplasm due to its interaction with the inhibitor IκB. The phosphorylation of IκB results in its degradation, followed by the translocation of NF-κB to the nucleus. NF-κB activates the expression of the cellular inhibitory protein FLICE (c-FLIP). FLIP blocks the interaction of pro-caspase -8 with FADD, resulting in an apoptotic inhibitory complex (AIC) [14,15,16]. On the other hand, caspase -8 can mediate Bid truncation. Truncated Bid (tBid) subsequently activates the mitochondrial pathway to amplify the apoptotic response [17] (Figure 1).

### 2.2. The Intrinsic or Mitochondrial Pathway

**Figure 2:**
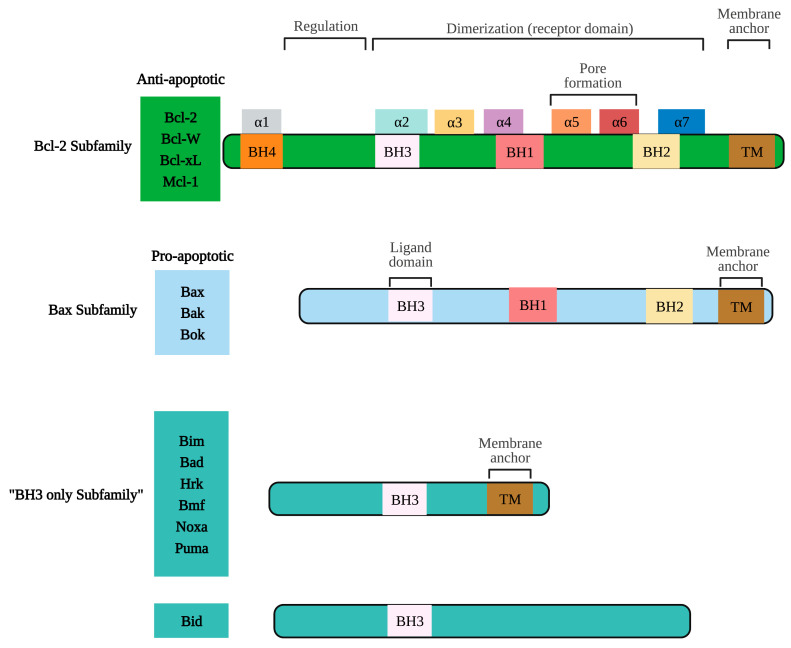
Different BH domains of Bcl-2 subfamilies members. Taken and modified from Jutinico et al. [18]. Bak: Bcl-2-antagonist killer 1; Bax: Bcl-2-associated X protein; Bcl: pro-apoptotic B-cell lymphoma; Bcl-xL: B-cell lymphoma-extra-large Bid: BH3- interacting domain death agonist; Bmf: Bcl-2 modifying factor; Bok: Bcl-2 related ovarian killer.

The intrinsic mitochondrial pathway is activated in response to extracellular stimuli such as drugs, radiation, oxidizing agents, toxins, xenobiotics, and intracellular stimuli such as hypoxia, extremely high concentrations of cytosolic calcium, intracellular reactive oxygen species (ROS), endoplasmic reticulum stress, mitochondrial translocation, genetic damage, activation of the p53 protein, guardian of the deoxyribonucleic acid (DNA) [17]. The pro-apoptotic and anti-apoptotic proteins of the Bcl-2 family regulate this pathway. The Bcl-2 family of proteins functions as cellular stress sensors that receive signals from the endoplasmic reticulum, cytoskeleton, mitochondria, and nucleus. The presence of Bcl-2 homology domains Bcl-2 (BH) characterizes this family. Four domains have been described: BH1, BH2, BH3, and BH4. The Bcl-2 protein family is classified into three subfamilies: 1) the Bcl-2 subfamily, with four BH domains: including Bcl-2, Bcl-extra-large, Bcl-w, Boo, A1, Mcl-1; 2) the Bax subfamily, which has three BH domains and possesses pro-apoptotic activities, has among its members Bax, Bok, and Bak itself, and 3) the BH3 “only” subfamily, which are pro-apoptotic proteins including Bik, Blk, Bad, Bid, Puma, and Noxa [18] (Figure 2).

**Figure 3:**
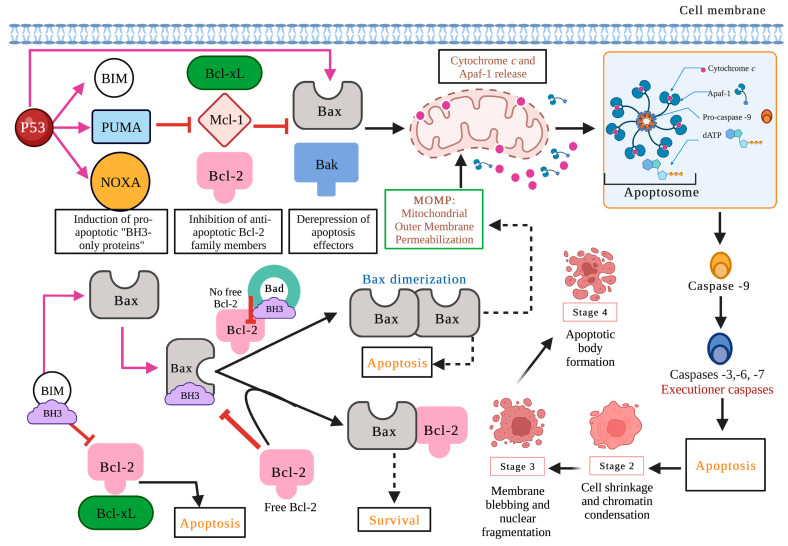
Mechanism of induction and inhibition of pro- and anti-apoptotic proteins by p53 and induction of apoptosis. Taken and modified from Aubrey et al. [20] and Giam et al. [21]. Activation and inhibition of pro-apoptotic and anti-apoptotic proteins, respectively, through the conformational change of Bax, facilitating the permeabilization of the mitochondrial membrane by pore formation, and, consequently, the release of cytochrome c from the mitochondria to the cytosol forming the apoptosome together with cytosolic Apaf-1 and pro-caspase-9, triggering regulated death. Appreciation of the morphological and biochemical changes of apoptosis. Bad: Bcl-2 agonist of cell death; Bak: Bcl-2 antagonist killer 1; Bax: Bcl-2-associated X protein; Bcl-xL: B-cell lymphoma-extra-large; Bcl-2: B-cell lymphoma 2; Bim: Bcl-2 interacting mediator of cell death; Caspase -3, -6, -7, -9: Cysteinyl aspartic acid-protease -3, -6, -7, -9; Mcl-1: Myeloid cell leukemia-1; PUMA: p53 upregulated modulator of apoptosis.

BH domains are essential for pro-apoptotic family members since they confer homo- and heterodimerization properties, which result in their activation or inactivation. This property is essential for pro-apoptotic BH3-only members who base much of their activity on the coupling to anti-apoptotic proteins to alter their activities [19]. The P53 protein stimulates pro-apoptotic proteins while inhibiting anti-apoptotic proteins in a transcription-dependent and -independent manner [20] (Figure 3). Activation of Bax and Bak is irreversible and inevitably culminates in cell death [21,22]. These proteins are translocated to the mitochondrial membrane inducing its permeability (MOMP: mitochondrial outer membrane permeabilization), releasing various protein components such as cytochrome *c*, apoptosis induction factor (AIF), caspase-activated DNA (CAD), second mitochondrial activator of caspases/direct inhibitor of apoptosis (IAP) binding protein with low PI (Smac/DIABLO) and high-temperature requirement protein A2 (Omi/HtrA2) to the cytoplasm. Smac/DIABLO and HtrA2/Omi promote apoptosis by IAPs [23].

Anti-apoptotic proteins maintain control of mitochondrial permeability, blocking the activity of pro-apoptotic proteins of that family. On the other hand, cytochrome c interacts with the cytosolic apoptotic protease activating factor-1 (Apaf)-1 protein, with dATP and pro-caspase -9 in the cytosol, forming the apoptosome. This protein complex activates caspase -9, which, in turn, activates effector caspases leading to cell death. AIF, independent of caspase, migrates to the nucleus during the late phase of apoptosis, causing DNA fragmentation, while CAD fulfills the same function but requires binding to caspase -3 [8]. A prominent substrate of caspase is poly (ADP-ribose) polymerase 1 (PARP-1), which is primarily activated by single-strand or double-strand DNA breaks [24]. PARP is cleaved explicitly by caspase -3, and this cleavage ensures that all self-destructing cells deactivate protective mechanisms such as DNA repair [25]. Biochemical and morphological features of apoptosis include DNA fragmentation, cytoplasmic contraction accompanied by externalization of phosphatidylserine (PS) to the outer layer of the plasma membrane, cell surface blisters, chromatin condensation, and the formation of apoptotic bodies [26].

### 2.3. Proteins of the Bcl-2 Family, P53, and Caspases in Carcinogenesis

It is important to emphasize that proteins of the Bcl-2 family are essential for the apoptotic process. It has been shown that the overexpression and deregulation of anti-apoptotic and pro-apoptotic proteins lead to cellular immortality and confer resistance to multiple drugs in tumor cells, avoiding apoptotic death [27]. On the other hand, it has been shown that the dysfunction of the tumor suppressor gene P53 could result in discontinuous and excessive cell proliferation that leads to carcinogenesis. P53 plays an essential role in the cell cycle and apoptosis. The fundamental role of P53 is its capacity to induce apoptosis in a transcription-dependent and transcription-independent manner. That suppressor gene behaves as an antagonist of the Bcl-2, inducing apoptosis in cancer cells mainly by direct transcriptional activation of pro-apoptotic proteins with BH3 domains only [20]. In various types of cancer, P53 loses its ability to activate pro-apoptotic proteins in a transcription-dependent and -independent manner. P53 can also directly interact with Bax, which stimulates the release of cytochrome c in the cytosol and, consequently, activates the apoptosis program [28].

A critical regulatory event in the apoptotic process is the activation of caspases. The relationship between caspase expression levels and cancer severity has been confusing and contradictory. It has been shown that the absence or deficient activity of caspase-8 generated primary neuroblastoma cells resistant to apoptosis in 75% of cases. In glioblastoma, it was reported that increased expression of caspase-8 was associated with a worse prognosis [14]. A stratification analysis revealed that in patients with oral tongue squamous cell carcinoma (OTSCC), a higher level of caspase -3 expression was associated with a better prognosis [29]. Caspases -6 and -7 are rarely mutated in human cancers. Caspase-6 mutations are associated with reduced caspase-6 expression in colon and gastric cancer. In contrast, somatic mutations in caspase -7 are associated with decreased apoptosis and have been detected in colon carcinoma, esophagus, and head/neck carcinoma [30]. The reasons for these contradictory results remain to be studied.

### 2.4. ROS, Ca^2+^, and Mitochondria in the Apoptotic Process

Oxidative stress is the imbalance between pro-oxidants and antioxidants that occurs in the body, which is eliminated by the body’s antioxidant defensive system [31]. It is involved in the development of different diseases, mainly cancer. Reactive oxygen species (ROS) play an essential role in oxidative stress and are crucial targets for the induction of apoptosis. An adequate level of intracellular ROS is vital for homeostasis and signaling associated with cell proliferation; however, an overproduction causes oxidative stress and degrades cellular proteins, lipids, and DNA, leading to the development of different diseases [32]. Mitochondria are the source and target of ROS generation [33]. Mitochondria are the energy supplier for cancer cells, considered one of the most critical organelles in cancer therapy [34]. Mitochondrial ROS are involved in numerous physiological processes and are called apoptosis inducers [35]. In addition, the progression of cancer to the metastatic stage associated with the metabolic reprogramming of cancer cells is linked to mitochondrial ROS [36].

On the other hand, it should be emphasized that mitochondria play an essential role in apoptosis. These organelles contain different pro-apoptotic compounds, such as cytochrome c, which can trigger the intrinsic pathway that leads to regulated cell death and/or molecules such as Smac/DIABLO that favor apoptosis by inhibiting IAPs [28]. Elevated ROS levels could trigger apoptosis by generating mitochondrial damage [37]. As a result of oxidative stress, pores along the mitochondrial membrane may oxidize or depolarize the mitochondrial membrane, releasing pro-apoptotic compounds into the cytoplasm and thus initiating the apoptotic program [37,38]. On the other hand, ROS plays a vital role in the balance of intra- and extracellular levels of Ca^2+^ [39]. Like ROS, Ca^2+^ maintains redox homeostasis and signaling events during normal physiological processes, so the interaction between ROS and Ca^2+^ may be bidirectional. Excess ROS directly damages plasma membrane fluidity and redox homeostasis, causing Ca^2+^ to disrupt the ion exchange balance between intra- and extracellular plasma membranes and mitochondria, releasing cytochrome *c* into the cytosol and, consequently, the activation of caspases -9 and -3. Antioxidants are the only therapeutic molecules with the most significant defensive role in cell protection since they can block oxidative stress by their ROS removal activity with low or no toxicity. However, under abnormal physiological conditions, endogenous antioxidants like glutathione or antioxidant defense enzymes (SOD: superoxide dismutase; CAT: catalase; GPx: glutathione peroxidase), although highly efficient, cannot protect the cell from the effects of ROS. Antioxidants play a vital role in cancer by counteracting the activity of ROS [40].

## 3. Pro-Apoptotic Activity of Bioactive Compounds from Seaweeds

The evasion of regulated cell death by different mechanisms is one of the most important molecular aspects in carcinogenesis because it contributes to cancer progression through tumor proliferation and metastasis. Therefore, inducing apoptosis became one of the most important goals in cancer therapy. Bioactive compounds activating apoptosis through the different signaling pathways can be used as chemotherapeutic agents and/or adjuvants. Therefore, the importance of different compounds is highlighted in Table 1. Carotenoids, phenolic compounds, phytosterols, glycoproteins, polysaccharides, and terpenes, extracted from macroalgae induced apoptosis in different cell lines of mammary adenocarcinoma (MCF-7, MDA-MB-231, and 4T1), prostate adenocarcinoma (DU-145 and PC3), lung adenocarcinoma (A549), carcinoma of the cervix (HeLa), colon carcinoma (ES2, Colo-205, HT-29, LoVo, OV90, and HCT116), gastric carcinoma (AGS), ovarian carcinoma (SKOV3), bladder carcinoma (5637), hepatocarcinoma (BEL-7402, HepG2, LM3, and SMMC-7721), leukemia (HL-60 and SKM-1), melanoma (A2058 and B16F10), and osteosarcoma (OSAS-2). The mechanisms involved include the regulation of death receptors of the extrinsic pathway, of the mitochondrial pathway, of the endoplasmic reticulum pathway, of ROS formation and Ca^2+^ release, positive and negative regulation of proteins of the Bcl-2 family, activation of initiating and effector caspases, biochemical and morphological changes of the apoptosis mechanism.

The American National Cancer Institute USA (NCI) established that the promising IC50 for botanicals/crude extracts should be lower than 20 μg/mL or 10 μM upon 48 or 72 h incubation [41]. The NCI considers an IC50 upper limit criteria of 30 μg/mL as a promising crude extract for purification [42]. On the other hand, Ayoub et al. (2014) consider an IC50 up to 100 μg/mL promising [43]. According to the results shown in Table 1, eight compounds were active below 7 μg/mL; 12 compounds were cytotoxic at concentrations from 8.7 to 25 μg/mL; four compounds with cytotoxic activity of 32.94 to 50 μg/mL, and six compounds with an IC50 from 55 to 100 μg/mL. These results demonstrated that these compounds of marine origins play an essential role in carcinogenesis, demonstrating their potential use as drugs and/or therapeutic adjuvants against death evasion, one of the most crucial challenges to be solved.

**Table 1:** Pro-apoptotic compounds extracted from macroalgae.

	Source	Compound	Cell Line	IC50	Treatment Time	Death Receptor Pathway	Mitochondrial Pathway	ROS/Ca^2+^ Activity	Bcl-2 Family Regulation	Caspases Activation	Others	Ref.
Carotenoids	*Gracilaria* sp.	β-carotene	HepG2	5.63 μg/mL	24 h	**✓**	**✓**		↑Bax ↓Bcl-2 ↑Bad	3	PARP cleavage	[44]
	*Ishige okamurae*	Fucoxanthin	B16F10	32.94 μg/mL					↓Bcl-xL	3 and 9	PARP cleavage	[45]
	Anhui University of Chinese Medicine		HeLa	0.65 μg/mL		**✓**			↑Bax ↓Bcl-2	3		[46]
	*Cladosiphon okamurus*	Fucoxanthinol	Saos-2	6.16 μg/mL					↓Bcl-2 ↓Bcl-xL	3, 8, and 9		[47]
	*Codium fragile*	Siphonaxanthin	HL-60	12.01 μg/mL		**✓**			↓Bcl-2	3		[48]
Phenolic Compounds	*Rhodomela confervoides*	^1^ BDDPM	BEL-7402	8.7 μg/mL						3 and 9	PARP cleavage	[49]
	*Ecklonia cava*	Dieckol	SKOV3	100 μg/mL		**✓**	**✓**	**✓**	↓Bcl-2	3, 8, and 9		[50]
	AKos Consulting & Solutions		A549	25 μg/mL						3, 8, and 9	DNA fragmentation Chromatin condensation	[51]
	Sigma-Aldrich (St. Louis, MO, USA)	Phloroglucinol	HT-29	50 μg/mL		**✓**	**✓**		↑Bax ↓Bcl-2 ↓Bcl-xL ↑Bad ↑Bid	3, 8, and 9	PARP cleavage	[52]
Phytosterols	*Codium fragile*	Clerosterol	A2058	61.90 μg/mL			**✓**		↑Bax ↓Bcl-2	3 and 9	DNA fragmentation	[53]
	Marine algae	Fucosterol	HL-60	14.19 μg/mL		**✓**	**✓**			3, 8, and 9	Presence of apoptotic bodies	[54]
	Sigma-Aldrich, (St. Louis, MO, USA)		HeLa	16.50 μg/mL			**✓**	**✓**			PS externalization	[55]
	Cayman Chemical Company (Ann Arbor, MI, USA)		ES2 OV90	25.75 µg/mL 21.21 µg/mL			**✓**	**✓**		3 and 9		[56]
	*Porphyra dentata*	Sterol fraction	4T1	48.3 μg/mL				**✓**				[57]
Glycoproteins	*Capsosiphon fulvescens*	^2^ CF-GP	AGS	3 μg/mL		**✓**	**✓**			3, 8, and 9		[58]
	*Codium decorticatum*	^3^ GLP	MCF-7	60 µg/mL							Nuclear fragmentation	[59]
			MDA-MB-231	55 μg/mL			**✓**	**✓**	↑Bax ↓Bcl-2	3 and 9	↑p53 Chromatin condensation Presence of apoptotic bodies PARP cleavage	[60]
	*Hizikia fusiformis*	^4^ HFGP	HepG2	25 μg/mL		**✓**	**✓**		↑Bax ↑Bad			[61]
Polysaccharides	*Enteromorpha intestinalis*	^5^ EI-SP	HepG2	98.4 μg/mL			**✓**		↑Bax ↓Bcl-2	3 and 9	PARP cleavage Chromatin condensation Nuclear fragmentation	[62]
	*Laurencia papillosa*	^6^ ESC	MDA-MB-231	50 µg/mL				**✓**	↑Bax ↓Bcl-2	3, 8, and 9	↑p53	[63]
	*Undaria pinnatifida*	Fucoidan	SMMC-7721	1000 μg/mL			**✓**	**✓**	↑Bax ↓Bcl-2	3, 8, and 9		[64]
	*Undaria pinnatifida*		PC-3	200 μg/mL		**✓**			↑Bax ↓Bcl-2	3, 8, and 9	PARP cleavage	[65]
	Sigma-Aldrich (St. Louis, MO, USA)		SKM-1	560 μg/mL		**✓**		**✓**		8 and 9		[32]
	*Undaria pinnatifida*		DU-145	750 µg/mL					↑Bax ↓Bcl-2	9	PARP cleavage	[66]
	Sigma-Aldrich (St. Louis, MO, U.S.A.)		5637	100 μg/mL			**✓**	**✓**	↑Bax ↓Bcl-2		↓c-myc	[67]
	Sigma-Aldrich (St. Louis, MO, U.S.A.)		MDA-MB-231	25 μg/mL			**✓**		↑Bax ↓Bcl-2 ↓Bcl-xL ↑Bid	3 and 9		[68]
	*Fucus vesiculosus*		LM3	400 µg/mL					↑Bax ↓Bcl-2	3, 8, and 9		[69]
	Sigma-Aldrich, (St. Louis, MO, USA)	Laminarin	LoVo	800 μg/mL			**✓**	**✓**		3 and 9		[70]
	*Laminaria japonica*		LoVo	800 μg/mL		**✓**		**✓**	↑Bid ↑tBid	3, 6, 7, and 9		[71]
	*Laminaria digitata*		ES2 OV90	2000 μg/mL			**✓**	**✓**			DNA fragmentation Endoplasmic reticulum stress	[72]
	*Ulva fasciata*	^7^ Se-PPC	A549	2.5 μg/mL		**✓**	**✓**	**✓**	↓Bcl-xL ↑Bax ↓Bcl-2 ↑Bid	3, 8, y 9	↑p53 Cell contraction Presence of Apoptotic bodies	[73]
Terpenes	*Stoechospermum marginatum*	^8^ DDSD	B16F10	3.7 μg/mL			**✓**	**✓**	↑Bax ↓Bcl-2	3 and 9	PS externalization DNA fragmentation Nuclear condensation	[74]
	*Laurencia dendroidea*	(-)-Elatol		2.52 µg/mL						2, 4, 6, and 8		[75]
	*Laurencia dendroidea*	+ (-) Obtusol	Colo-205	1.35 µg/mL						6		[75]
	*Pterocladiella capillacea*	Mertensene	HT-29	90 μg/mL		**✓**				3	PARP cleavage	[76]
	*Sargassum macrocarpum*	^9^ TTB	MDA-MB-231, A549, and HCT116	25.35, 20.28, and 19.86 μg/mL				**✓**	↓Bcl-2	3	PARP cleavage	[77]

^1^ BDDPM: Bis (2,3-dibromo-4,5-dihydroxy-phenyl)-methane; ^2^ CF-GP: Glycoprotein from *Capsosiphon fulvescens*; ^3^ GLP: Glycoprotein from *Codium decorticatum*; ^4^ HFGP: Glycoprotein from *Hizikia fusiformis*; ^5^ EI-SP: Sulphate polysaccharide from *Enteromorpha intestinalis*; ^6^ ESC: Extract sulfated carrageenan; ^7^ Se-PPC: Selenium-Containing Polysaccharide-Protein Complex; ^8^ DDSD: 5(R),19-diacetoxy-15, 18 (R and S), dihydro spata-13, 16(E)-diene; ^9^ TTB: Tuberatolide B; A2058: metastasic melanoma; A549: lung cancer; AGS: gastric cancer; Bad: Bcl-2 agonist of cell death; Bax: Bcl-2-associated X protein; Bcl-xL: B-cell lymphoma-extra-large; Bcl-2: B-cell lymphoma 2; BEL-7402: hepatocarcinoma; Bid: BH3- interacting domain death agonist; B16F10: melanoma; Caspase -2, -3, -4, -6, -7, -8, -9: Cysteinyl aspartic acid-protease -2, -3, -4, -6, -7, -8, -9; Colo-205: colorectal cancer: DNA: Deoxyribonucleic Acid; Du-145: prostate cancer; ES2: colorectal cancer; HepG2: hepatocarcinoma; HL-60: leukemia; HT-29: colorectal cancer; LM3: hepatocarcinoma; LoVo: colorectal cancer; MCF-7: breast adenocarcinoma; MDA-MD-231: breast adenocarcinoma; OV90: colorectal cancer; PARP: poly (ADP-ribose) polymerase; PC-3: prostate cancer; Saos-2: osteosarcoma; SKM-1: leukemia; SKOV3: ovarian carcinoma; SMMC-7721: hepatocarcinoma; tBid: Truncated Bid; 4T1: breast adenocarcinoma; 5637: bladder cancer.

### 3.1. Carotenoids

**Figure 4:**
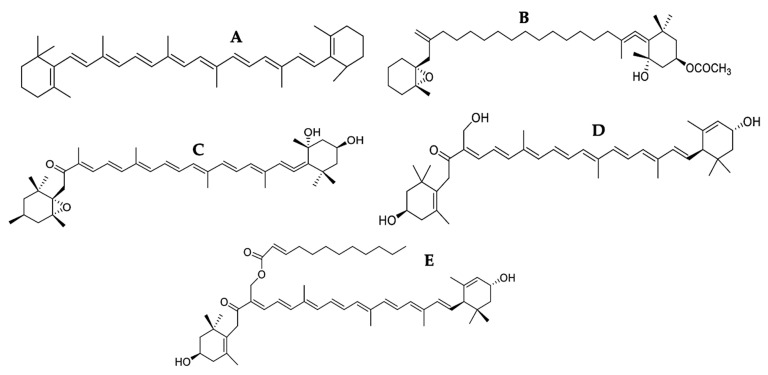
Chemical structure of carotenoids. (**A**): β-carotene; (**B**): Fucoxanthin; (**C**): Fucoxanthinol; (**D**): Siphonaxanthin; (**E**): Siphonein.

**Figure 5:**
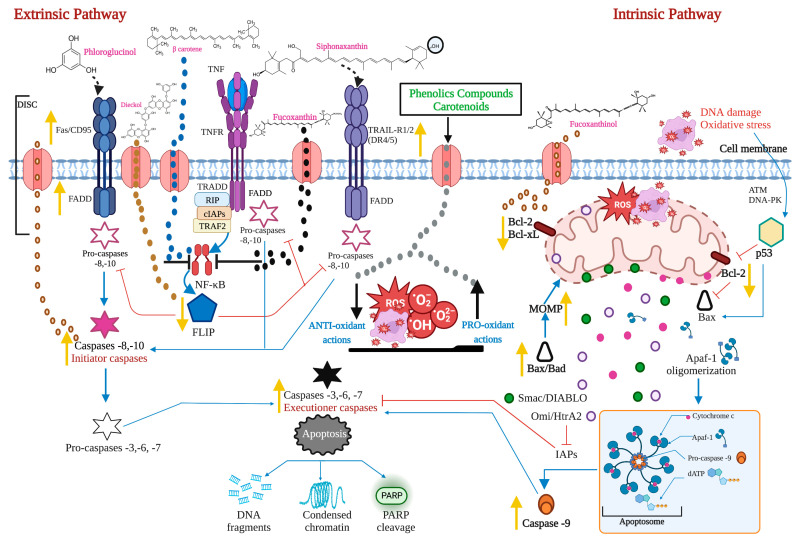
Possible mechanism of action of phenolic compounds and carotenoids extracted from macroalgae inducing apoptosis. It represents the mechanism of action of some compounds such as phloroglucinol, dieckol, siphonaxanthin, fucoxanthin, and fucoxanthinol in the extrinsic and intrinsic pathway of apoptosis, modulating target molecules involved in them. Representation of the pro-oxidant action of phenolic compounds and carotenoids generates an overproduction of intracellular ROS, which causes mitochondrial dysfunction allowing the release of pro-apoptotic compounds to the cytosol, triggering apoptosis. Yellow arrows indicate positive or negative regulation of crucial proteins by these phenolic compounds and carotenoids. Apaf-1: apoptotic protease activating factor-1; ATM: Ataxia-telangiectasia-mutated; Bax: Bcl-2-associated X protein; Bcl-2: B-cell lymphoma; Bcl-xL: B-cell lymphoma-extra-large; Caspase -3, -6, -7, -8, -9, and 10: Cysteinyl aspartic acid-protease-3, -6, -7, -8, -9, and -10; DNA-PK: DNA-dependent protein kinase; DR 4/5: Death receptor 4/5; FADD: Fas-associated death domain; Fas: FLIP: (FADD-like IL-1β-converting enzyme)-inhibitory protein; HtrA2: High-temperature requirement protein A2; IAP: Inhibitors of Apoptosis Proteins; MOMP: Mitochondrial Outer Membrane Permeabilization; NF-κB: nuclear factor kappa-light-chain-enhancer of activated B cells; PARP: poly (ADP-ribose) polymerase; RIP: Receptor interacting protein; SMAC/DIABLO: Second mitochondrial activator of caspases/direct IAP binding protein with low PI; TNF: Tumor necrosis factor; TNFR1: TNF receptor 1; TRADD: TNF receptor-associated death domain; TRAF2: TNFR-associated factor 2; TRAIL: TNF-related apoptosis-inducing ligand.

Carotenoids are fat-soluble tetraterpenoid pigments divided into xanthophylls containing oxygen and carotene, pure hydrocarbons [78]. Xanthophylls appear to extend within the phospholipid bilayer with their polar functional groups. At the same time, nonpolar carotenes are found inside the biomembrane’s hydrophobic nucleus, altering the packing of acyl phospholipid chains complying with pro-oxidant actions in lipid peroxidation [79]. Phenolic compounds such as carotenoids can act as pro-oxidant molecules triggering ROS-mediated apoptosis in cancer cells that produce elevated levels of ROS. It has been shown that the coadministration of carotenoids with ROS-inducing cytotoxic drugs (doxorubicin, cisplatin, Taxol, and paclitaxel) minimizes the adverse effects of these chemotherapeutic agents on normal cells. They act as antioxidants without interfering with the cytotoxic, pro-oxidant anticancer drugs [80]. Different studies have indicated that the antioxidant effect of carotenoids depends on their structure, location, or site of action [81]. The chemical structure of some carotenoids with pro-apoptotic activity is shown in Figure 4, and their possible mechanism of action is in Figure 5.

β-carotene (**A**)-induced apoptosis in HepG2 cells with an IC50 of 10.5 µM (3.18 μg/mL) and inhibited NF-κB. This compound increased and decreased the expression of Bax and Bcl-2, respectively, decreased the mitochondrial membrane potential of the intrinsic pathway, activated caspase -3, and triggered the cleavage of PARP [44] (Figure 5). NF-κB induces cell proliferation, metastasis, and resistance to cancer therapy and suppresses apoptosis by induction of anti-apoptotic proteins and suppressing pro-apoptotic genes. In addition, this transcription factor inhibits p53-induced apoptosis by downregulating p53 expression [16]. Therefore, inhibiting NF-κB by β-carotene (**A**) was essential to induce apoptosis. On the other hand, cancer cells upregulate some transcription factors and enzymes that help protect and survive cells to counteract intracellular oxidative stress. Nrf-2 (nuclear factor E2-related factor), one of those transcription factors controlling the cell’s antioxidant cellular defense system, was downregulated by β-carotene (**A**). The β-carotene (**A**) reduced the expression of superoxide dismutase-2 (SOD-2), which is one of the regulatory genetic targets of Nrf-2 [44]. SOD-2 is an antioxidant enzyme that removes excess mitochondrial ROS converting it into H_2_O_2_ and O_2_, thus playing a pivotal role in cell protection. In addition, overexpression of SOD-2 confers resistance to mitochondrial permeability transition inducers [33]. In contrast, the overproduction of mitochondrial ROS leads to the release of apoptosis-inducing protein compounds. In that study, the concentration of β-carotene (**A**) did not alter intracellular levels of ROS but rather downregulated the essential antioxidant enzymes that maintain redox homeostasis in cancer cells. This result confirms that β-carotene (**A**) can modify t the intracellular antioxidant status [40]. Therefore, the negative regulation of these enzymes was vital in the induction of apoptosis by the mitochondrial pathway.

With an IC50 of 32.94 μg/mL, fucoxanthin (**B**) extracted from *Ishige okamurae* induced apoptosis in B16F10 cells by upregulating and downregulating the expression of Bax and Bcl-xL, respectively, and consequently activating caspases-9 and -3, leading to cleavage of PARP [45]. Likewise, fucoxanthin (**B**) induced apoptosis in HeLa cells with an IC50 of 0.65 μg/mL. This compound inhibited NF-κB, increased and decreased the expression of Bax and Bcl-2, respectively, and activated caspase-3, generating apoptosis [46] (Figure 5). Fucoxanthin (**B**), a xanthophyll with an allenic bond and 5,6-monoepoxide, is one of the most abundant carotenoids, contributing to more than 10% of the estimated total carotenoid production in nature [82,83]. It has antioxidant, anticancer, antiobesity, and antidiabetic properties [81]. NF-κB is a protein complex that controls DNA transcription, and its activation generates cell survival, promoting cancer cell growth and inhibiting apoptosis [46]. In addition, the NF-κB pathway is also an oxidative response pathway [40]. Studies have shown that carotenoids can inhibit this pathway induced by oxidative stress by adding electrophilic groups to cysteine residues to IκB subunits [84]. Fucoxanthin had a better cytotoxic effect on HeLa with an IC50 of 0.65 µg/mL versus an IC50 of 32.94 µg/mL in B16F10 (melanoma), inducing apoptosis through both pathways only in HeLa. These results reflect the susceptibility of HeLa to treatment, the effectiveness of the treatment, and the resistance of B16F10. Fucoxanthin is one of the most studied carotenoids; however, more studies are needed because it is a compound with potent cytotoxicity on different tumor lines. Fucoxanthinol (**C**), the deacetylated metabolite of fucoxanthin (**B**), generated apoptosis in Saos-2 cells, with an IC50 of 6.16 µg/mL, activating caspase -8 of the extrinsic pathway, negatively regulating the expression of Bcl-2, Bcl-xL, and XIAP, activated caspase-9 of the intrinsic pathway leading to activation of caspase-3 [47] (Figure 5).

Apoptosis induction and decreased cell viability of HL-60 cells were reported after six hours of treatment with siphonaxanthin (**D**), extracted from *Codium fragile*, by upregulating TRAIL-R2/DR5 from the extrinsic pathway, decreasing Bcl-2 expression and activating caspase -3 with an IC50 of 12.01 µg/mL [48]. The apoptosis inducer ligand TRAIL, a TNF (Tumor necrosis factor) family member, is a type II transmembrane protein with an extracellular domain that can be cleaved to take its biologically active soluble form, initiates apoptosis through its interaction with the DR4 and DR5 death receptors. Not all cancer cells are susceptible to TRAIL-mediated apoptosis. DR4 and DR5 receptors not only trigger apoptosis in TRAIL-sensitive cells but also activate survival pathways in TRAIL-resistant tumor cells, and these cells escape destruction by the immune system [85]. Therefore, it is vital to find compounds, design effective therapeutic strategies for TRAIL-resistant cancers, and improve drugs for TRAIL-sensitive cancers. Siphonaxanthin (**D**) contains an additional hydroxyl group on the 19th carbon atom, unlike other carotenoids such as fucoxanthin (**B**) and siphonein (**E**). This additional hydroxyl group appears to contribute to the apoptosis-inducing effect because siphonein, an esterified form of siphonaxanthin, had a reduced inhibitory effect on cell viability [86]. Due to the characteristics of siphonaxanthin (**D**), it has likely bound to the transmembrane receptor (TRAIL-R2/DR5) of the death ligand TRAIL, which activates the extrinsic apoptotic pathway (Figure 5). These results confirm the pro-apoptotic effect of carotenoids through their antioxidant and pro-oxidant action.

### 3.2. Phenolic Compounds

**Figure 6:**
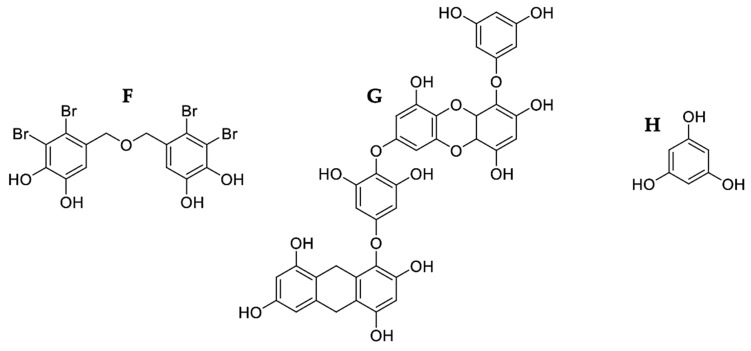
Chemical structure of phenolic compounds extracted from macroalgae with apoptotic activity. (**F**): BDDPM; (**G**): Dieckol; (**H**): Phloroglucinol.

Phenolic compounds consist of at least one aromatic phenolic ring joined with one or more hydroxyl groups. Those aromatic rings allow them to capture free radicals and chelate metal ions [87] and eliminate ROS, donating H atoms or transferring an electron from hydroxyl groups followed by protonation. Removing oxidants is essential to control cancer; therefore, phenolic compounds of seaweed are valuable as a natural source of antioxidant agents. Free radicals against membrane lipids, proteins, enzymes, DNA, and RNA, play a fundamental role in cancer. Therefore, antioxidants benefit human health by preventing free radical damage [88]. It should be noted that the antioxidant activity of phenolic compounds is related to other bioactivities, such as anti-inflammatory, antitumor, hypocholesterolemic, anticoagulant, antiviral, and antimicrobial activities [89]. Some phenolic compounds’ possible mechanism of action is shown in Figure 5, and their chemical structure is in Figure 6.

BDDPM (**F**), a bromophenol extracted from *Rhodomela confervoides*, activated caspases -9 and -3 of BEL-7402 cells at an IC50 of 8.7 μg/mL, culminating in the apoptotic process with PARP cleavage [49]. Dieckol (**G**), a phlorotannin extracted from *Ecklonia cava*, downregulated the expression of cFLIP and activated caspase-8 of the extrinsic pathway, respectively, increased intracellular ROS, causing mitochondrial dysfunction, decreased the expression of Bcl-2 and XIAP, caused the release of cytochrome *c* and activated caspases-9 and -3 of SKOV3 cells, with an IC50 of 100 μg/mL [50] (Figure 5). cFLIP is a caspase inhibitor of the extrinsic pathway. Its increased expression has been reported in different types of cancer [8]. Therefore, its inhibition by dieckol (**G**) is essential to induce apoptosis in this pathway. At an IC50 of 25 μg/mL, Dieckol activated caspases-8 and -9 of the extrinsic and intrinsic pathways, leading to caspase-3 activation, culminating the apoptotic process with DNA fragmentation and A549 cell chromatin condensation [51] (Figure 5). Dieckol had a better cytotoxic effect on A549 with an IC50 of 25 µg/mL versus an IC50 of 100 µg/mL in SKOV3, inducing apoptosis through both pathways. These results reflect the susceptibility of A549 to treatment and resistance of SKOV3. The antiproliferative, antidiabetic, anti-HIV, anti-allergic, anti-inflammatory, and antioxidant activity of phlorotannins has been reported [81]. Its high antioxidant activity is due to its highly hydrophilic components and the presence of -OH groups that can form hydrogen bonds with water [40].

With an IC50 of 50 μg/mL, phloroglucinol (**H**), the basic unit of phlorotannins, upregulated the expression of Fas, FADD, caspase -8, and Bid of the extrinsic pathway. On the other hand, it increased the expression of Bax and Bad and decreased the expression of Bcl-2 and Bcl-xL, activating the caspases -9 and -3 in HT-29 cells, generating apoptosis by both pathways [52] (Figure 5). The Bcl-2 proteins family is the central regulator of intrinsic apoptosis. Bcl-2 binds to pro-apoptotic members such as Bax, preventing the formation of mitochondrial pores and thus releasing apoptosis-inducing compounds. In contrast, increased Bax expression induces cell death [63]; therefore, regulating the Bcl-2 family by phloroglucinol was vital in inducing apoptosis via the intrinsic pathway. These results demonstrated the fundamental role of phenolic compounds as antioxidants in cancer treatment.

### 3.3. Phytosterols

**Figure 7:**
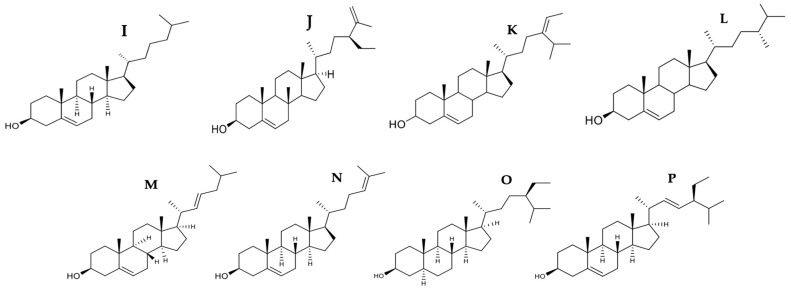
Chemical structure of phytosterols extracted from macroalgae. (**I**): Cholesterol; (**J**): Clerosterol; (**K**): Fucosterol; (**L**): Campesterol; (**M**): 22-dehydrocolesterol; (**N**): Desmosterol; (**O**): β-sitosterol; (**P**): Stigmasterol.

**Figure 8:**
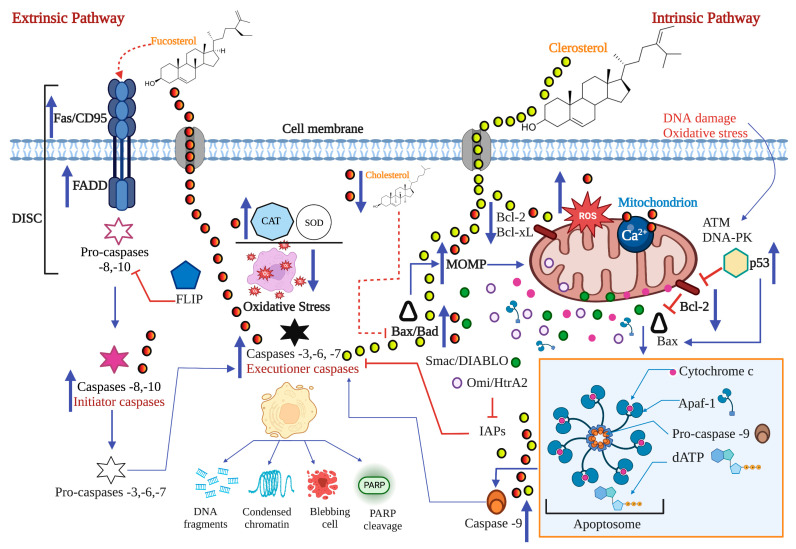
Possible mechanism of action of phytosterols extracted from macroalgae inducing apoptosis. It represents the mechanism of action of some compounds, such as clerosterol and fucosterol, in the extrinsic and intrinsic apoptosis pathway, modulating the target molecules involved. High mitochondrial cholesterol content has been found in different tumors resulting in partial or ineffective oligomerization of Bax that could contribute to apoptotic resistance; due to that, the capacity of phytosterols to decrease cholesterol content is crucial. Violet arrows indicate positive or negative regulation of crucial proteins by such phytosterols. Apaf-1: apoptotic protease activating factor-1; ATM: Ataxia-telangiectasia-mutated; Bax: Bcl-2-associated X protein; Bcl-2: B-cell lymphoma; Bcl-xL: B-cell lymphoma-extra-large; Bid: BH3- interacting domain death agonist; Caspase -3, -6, -7, -8, -9, -10: Cysteinyl aspartic acid-protease -3, -6, -7, -8, -9, -10; CAT: catalase; DNA-PK: DNA-dependent protein kinase; DR 4/5: Death receptor 4/,5; FADD: Fas-associated death domain; FLIP: (FADD-like IL-1β-converting enzyme)-inhibitory protein; HtrA2: High-temperature requirement protein A2; IAP: Inhibitors of Apoptosis Proteins; MOMP: Mitochondrial Outer Membrane Permeabilization; NF-κB: nuclear factor kappa-light-chain-enhancer of activated B cells; PARP: poly (ADP-ribose) polymerase; RIP: Receptor interacting protein; SMAC/DIABLO: Second mitochondrial activator of caspases/direct IAP binding protein with low PI; SOD: superoxide dismutase; tBid: Truncated Bid.

Phytosterols are sterols with a structure like cholesterol (**I**) with additional ethyl or methyl group in the side chain [81]. Its consumption is related to a lower risk of cancer by 20% [90]. Lipids play an important role in different mechanisms of apoptosis and Bax regulation. At high concentrations or accumulation of cholesterol in the mitochondria, it can behave as a Bax inhibitor preventing its activation or oligomerization by decreasing its membrane binding capacity [91]. High mitochondrial cholesterol content has been found in different tumors resulting in partial or ineffective oligomerization of Bax that could contribute to apoptotic resistance [92]. Regarding the above, the use of statins becomes essential. However, the side effects, such as rhabdomyolysis, myopathy, and elevated creatine kinase, caused by them are questionable [93]. Therefore, phytosterols are an alternative because they are appreciated for their ability to lower cholesterol [94]. The chemical structure of some phytosterols with pro-apoptotic activity is shown in Figure 7 and Figure 8, respectively.

Clerosterol (**J**) extracted from *Codium fragile* induced A2058 cell apoptosis by up and down-regulation of Bax and Bcl-2 expression, activating caspases -9 and -3 at an IC50 of 61.90 µg/mL [53]. It is likely that clerosterol (**I**), an apolar compound, can penetrate the cell directly through the lipid bilayer of the plasma membrane. Once inside the cell, clerosterol (**J**) can stimulate Bax by lowering the level of mitochondrial cholesterol and facilitating its total oligomerization or its union with the mitochondrial membrane causing the loss of MOMP in such a way that this apoptosis pathway is activated (Figure 8).

Fucosterol (**K**) positively regulated the expression of extrinsic pathway proteins such as Fas, FasL, FADD, and caspase -8, decreased MMP, and activated caspases -9 and -3 with an IC50 of 14.19 µg/mL in HL-60 cells [54]. Studies showed that fucosterol could cross the cell membrane to reach various intracellular targets [40]. Fucosterol (**K**) also increased intracellular ROS, causing loss of mitochondrial membrane potential in HeLa cells at an IC50 of 16.15 µg/mL [55]. Fucosterol increased the concentration of ROS and intracellular calcium. It caused mitochondrial membrane potential (MMP) loss in ES2 cells with an IC50 of 25.75 µg/mL versus 21.21 µg/mL in OV90 cells [56]. One study demonstrated that fucosterol (**K**) attenuated oxidative stress by upregulating antioxidant enzymes such as SOD and CAT by activating Nrf-2 [95]. In carcinogenesis, mitochondrial malfunction increases mitochondrial calcium, which can affect the release of apoptotic factors [56]. These results showed that fucosterol induced apoptosis through the production of ROS, overloading mitochondrial calcium concentration, thereby allowing the release of apoptosis-inducing compounds from the mitochondria to the cytosol. It is important to note that the change in mitochondrial membrane permeability is considered one of the most significant events in the apoptotic process. On the other hand, fucosterol (**K**) is a sterol abundant in seaweed that presents antioxidant, anticancer, antidiabetic, anti-inflammatory, anti-obesity, and mainly hypocholesterolemic activity [54,96]. Therefore, it is likely that this property of fucosterol (**K**) favors the activation of Bax, consequently, cell death [91] (Figure 8).

The effect of fucosterol has been studied on different tumor cell lines, such as colon carcinoma (ES2 and OV90), carcinoma of the cervix (HeLa), and leukemia (HL-60). Fucosterol had a better cytotoxic effect on HL-60 with an IC50 of 14.19 µg/mL inducing apoptosis through both signaling pathways, against an IC50 between 21.21–25.75 µg/mL for the other lines, with apoptosis induced only through the intrinsic pathway. These results reflect the vulnerability of HL-60 to treatment and the resistance of others. Further studies of fucosterol are needed because it is a compound with excellent pharmacology potential and an IC50 below 26 µg/mL (potent cytotoxicity) in different tumor lines.

A fractionated sterol extracted from *Porphyra dentata* decreased the cell viability of 4T1 cells with an IC50 of 48.3 µg/mL at 48h and caused the externalization of phosphatidylserine to the plasma membrane. On the other hand, 100 µL (5 × 10^6^ cells/mL) of suspension of these cells were inoculated to mice subcutaneously in the breast fat pads to be injected intraperitoneally with 20 µL of sterol fractionated in doses of 5, 10, and 25 mg/kg/day. Doses of 10 and 25 mg/kg/day inhibited tumor nodule growth, increased the survival rate of mice, and significantly decreased ROS activity [57]. Several sterol components have been identified in algae of the genus *Porphyra*, including campesterol (**L**), cholesterol (**I**), 22-dehydrocholesterol (**M**), desmosterol (**N**), fucosterol (**K**), β-sitosterol (**O**), and stigmasterol (**P**) [97,98]. They have antitumor properties [99]. According to Kazlowska et al., the identified compounds from this sterol component by HPLC-ELSD (High-Performance Liquid Chromatography Evaporative Light Scattering Detector) were cholesterol (**I**), campesterol (**L**), and β-sitosterol (**O**), with relative weight percentages of 15, 30, and 55%, respectively [57]. Phytosterols exhibit anticancer activities by inhibiting carcinogen production, cancer cell proliferation, angiogenesis, invasion and metastasis, and induction of apoptosis [100]. Therefore, the authors suggest that the antiproliferative activity, the apoptotic-necrotic effect on cells, and the decrease in ROS are probably due to the presence of β-sitosterol (**O**) and campesterol (**L**) [57].

In summary, these results demonstrated the apoptotic activity of phytosterols, which could be used as therapeutic adjuvants to obtain synthetic drugs against cancer.

### 3.4. Glycoproteins

**Figure 9:**
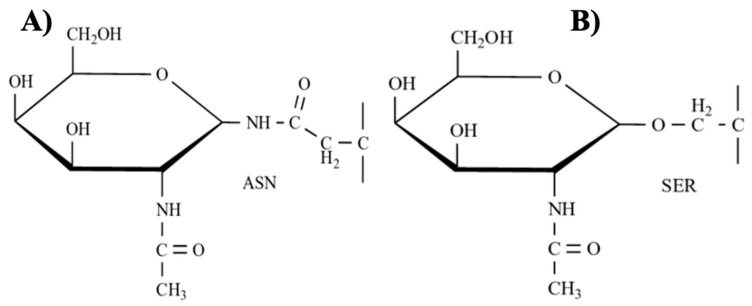
Chemical structure of *N*- and *O*-linked glycosyl bonds of glycoproteins. (**A**): *N*-Linked and (**B**): *O*-Linked.

**Figure 10:**
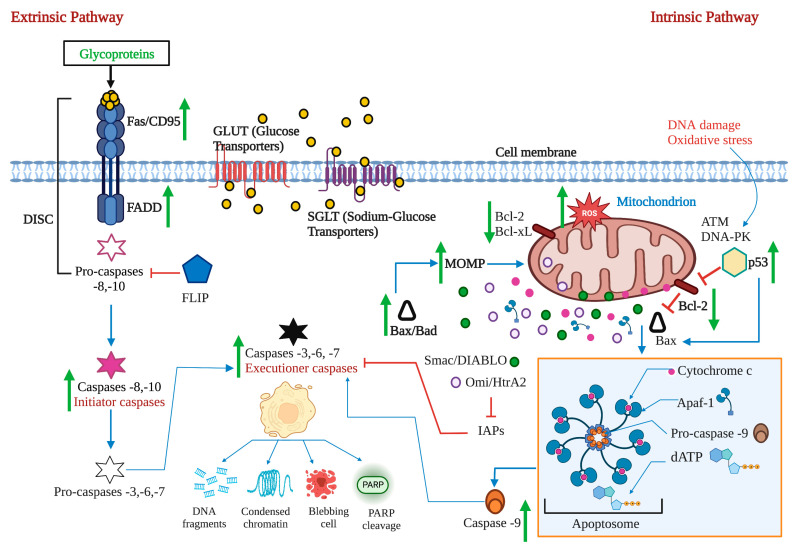
Possible mechanism of action of glycoproteins extracted from macroalgae inducing apoptosis. The mechanism of action of glycoproteins in the extrinsic and intrinsic apoptosis pathway is represented, modulating target molecules involved in them. The glycoproteins generate an overproduction of intracellular ROS, which causes mitochondrial dysfunction allowing the release of pro-apoptotic compounds to the cytosol, triggering apoptosis. Green arrows indicate positive or negative regulation of crucial proteins by these glycoproteins. Apaf-1: apoptotic protease activating factor-1; ATM: Ataxia-telangiectasia-mutated; Bax: Bcl-2-associated X protein; Bcl-2: B-cell lymphoma; Bcl-xL: B-cell lymphoma-extra-large; Caspase -3, -6, -7, -8, -9, -10: Cysteinyl aspartic acid-protease -3, -6, -7, -8, -9, -10; DNA-PK: DNA-dependent protein kinase; FADD: Fas-associated death domain; FLIP: (FADD-like IL-1β-converting enzyme)-inhibitory protein; HtrA2: High-temperature requirement protein A2; IAP: Inhibitors of Apoptosis Proteins; MOMP: Mitochondrial Outer Membrane Permeabilization; PARP: poly (ADP-ribose) polymerase; SMAC/DIABLO: Second mitochondrial activator of caspases/direct IAP binding protein with low PI.

Glycoproteins are proteins covalently modified with glycans in specific amino acid residues by glycosylation, wherein glycans are conjugated to peptide chains by *N*- and *O*-glycosyl bonds [59] (Figure 9). They are biomolecules in cell membranes that act as transport proteins essential for cell-cell signaling, cell-matrix interaction, energy storage, adhesion, and intracellular trafficking [101]. Abnormal glycosylation of proteins has been reported to involve the progression of diseases such as cancer [102]. The release of these aberrantly glycosylated proteins reflects the abnormal states of malignant cells [101]. Systematic and especially site analysis of glycoproteins located on the surface of the cell membrane is complicated due to the heterogeneity of glycans, the low abundance of many surface glycoproteins, and the requirement for effective methods to separate them [102]. Therefore, abnormal protein glycosylation patterns are crucial for finding biomarkers and therapeutic interventions against cancer [103]. Consequently, glycoproteins extracted from macroalgae play an essential role in carcinogenesis because they possess anticancer, antioxidative, antiproliferative, hepatoprotective, and anti-inflammatory properties [59]. Figure 10 shows the possible mechanism of action of different glycoproteins extracted from macroalgae with pro-apoptotic activity.

A glycoprotein extracted from *Capsosiphon fulvescens* (CF-GP) increased the expression of Fas, FADD, and caspase -8 of the extrinsic pathway, causing the release of cytochrome c into the cytosol, and activated caspases -9 and -3, from the intrinsic pathway of AGS cells with an IC50 of 3 μg/mL, triggering apoptosis [58]. Likewise, a glycoprotein extracted from *Hizikia fusiformis* (HFGP) increased the expression of Fas and FADD of the extrinsic pathway, increased the expression of Bax and Bad, and caused the release of cytochrome c to the cytosol, inducing apoptosis of HepG2 cells by the intrinsic pathway, at an IC50 of 25 μg/mL [61]. It is important to emphasize that glycosylation mainly influences the extrinsic apoptotic program involving the TRAIL and Fas death receptors (CD95/APO-1). The latter has two N-glycosylation sites in N136 and N118 that moderately affect the apoptosis induced by it. The addition of sialic acids by ST6Gal-1 in a α2-6 bond to Fas N-glycans has been shown to protect the cell against Fas-mediated apoptosis in colon carcinoma cells. The α2-6 sialylation of Fas prevents FasL-induced apoptosis by low activation of caspases -8 and -3, blocking the FADD association with the cytoplasmic domains of Fas and inhibiting Fas internalization [104]. N-deglycosylation of Fas reduces the activation rate of pro-caspase -8 without impact on death-inducing signaling complex (DISC) formation or FADD recruitment [105]. CF-GP and HFGP likely stimulated the Fas death receptor, a transmembrane glycoprotein with cysteine-rich extracellular domains, transmitting the apoptotic signal via the FADD adapter, which converts the caspase -8 zymogen into its active form, triggering the apoptotic program. Stimulating Fas and preventing its N-glycosylation is essential because the latter inhibits apoptotic signaling that leads to cell death.

A glycoprotein (GLP) extracted from *Codium decorticatum* induced apoptosis to MDA-MB-231 cells at an IC50 of 55 μg/mL by decreasing and increasing the expression of Bcl-2 and Bax, respectively, increased the level of ROS, which caused the loss of mitochondrial membrane potential thus releasing cytochrome c to the cytosol allowing the apoptosome to form resulting in the activation of caspase -9 and consequently caspase -3 [60] (Figure 10). Likewise, it increased the expression of p53 and caused the cleavage of PARP-1, chromatin condensation, and apoptotic body formation. Likewise, this same glycoprotein induced apoptosis to MCF-7 cells at an IC50 of 60 μg/mL, resulting in nuclear fragmentation [59]. GLP is composed of 36.24 and 63.76% carbohydrates and proteins, respectively. It consists of four monosaccharides, namely rhamnose (38%), galactose (30%), glucose (26%), mannose (6%), and 13 amino acids, of which five are essential. Due to the characteristics of GLP, it has likely entered the cell through the hydrophobic section of the peptide or transporter proteins of SGLT (sodium-glucose transporters) and GLUT (glucose transporters) by change of conformation (Figure 10). Once inside the cell, it is suggested that GLP dephosphorylated Bax, causing a conformational change that allows its dimerization and thus deactivates or inhibits the expression of Bcl-2 or Bcl-xL to translocate to the mitochondrial membrane compromising its membrane and executing the apoptotic program.

According to [59], studies on macroalgae glycoproteins with anticancer properties are minimal, so they should become a new object of study. In summary, these results confirmed the apoptotic activity of glycoproteins, which could be used as therapeutic adjuvants in cancer treatment.

### 3.5. Polysaccharides

**Figure 11:**
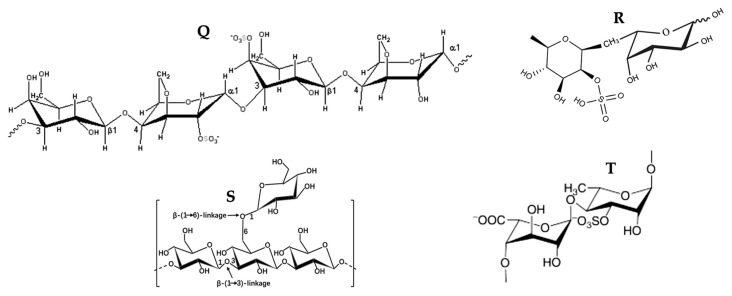
Chemical structure of polysaccharides extracted from macroalgae with apoptotic activity. (**Q**): Carrageenan; (**R**): Fucoidan; (**S**): Laminarin; (**T**): Ulvan.

**Figure 12:**
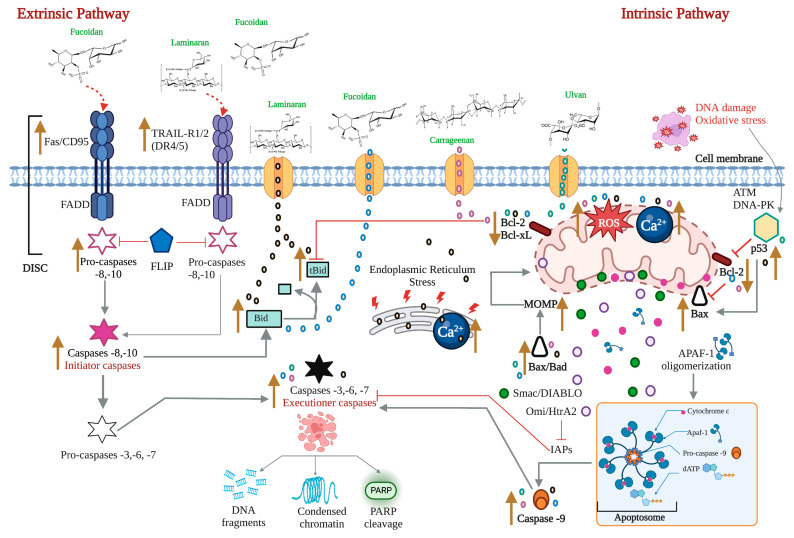
Possible mechanism of action of polysaccharides extracted from macroalgae inducing apoptosis. It represents the mechanism of action of some compounds such as fucoidan, laminarin, carrageenan, and ulvan in the extrinsic and intrinsic apoptosis pathway, modulating target molecules involved in them. The polysaccharides generate an overproduction of ROS and Ca^2+^ intracellular, which causes mitochondrial dysfunction allowing the release of pro-apoptotic compounds to the cytosol, triggering apoptosis. Brown arrows indicate positive or negative regulation of crucial proteins by such polysaccharides. Apaf-1: apoptotic protease activating factor-1; ATM: Ataxia-telangiectasia-mutated; Bax: Bcl-2-associated X protein; Bcl-2: B-cell lymphoma; Bcl-xL: B-cell lymphoma-extra-large; Bid: BH3- interacting domain death agonist; Caspase -3, -6, -7, -8, -9, -10: Cysteinyl aspartic acid-protease -3, -6, -7, -8, -9, -10; DNA-PK: DNA-dependent protein kinase; DR 4/5: Death receptor 4/5; FADD: Fas-associated death domain; Fas: FLIP: (FADD-like IL-1β-converting enzyme)-inhibitory protein; HtrA2: High-temperature requirement protein A2; IAP: Inhibitors of Apoptosis Proteins; MOMP: Mitochondrial Outer Membrane Permeabilization; NF-κB: nuclear factor kappa-light-chain-enhancer of activated B cells; PARP: poly (ADP-ribose) polymerase; RIP: Receptor interacting protein; SMAC/DIABLO: Second mitochondrial activator of caspases/direct IAP binding protein with low PI; tBid: Truncated Bid.

Polysaccharides are carbohydrates composed of long chains of monosaccharides or disaccharides linked by glycosidic bridges. Their diversity in structure and property is due to reactive groups, molecular weights, and variable chemical composition [106]. Alginates, agar, carrageenan, ulvans, fucoidan, laminarin, porphyrins, and agarose are produced by seaweeds. These polysaccharides are essential because they possess several pharmacological activities, including antitumor activity [107]. Moreover, different studies have demonstrated the antioxidant activity in vitro of polysaccharides, eliminating free radicals and the chelating capacity of metals. This antioxidant activity is highly related to its degree of sulfation, relative molecular mass, dominant sugar type, and glycosidic branching [81]. The chemical structure of some polysaccharides with pro-apoptotic activity is shown in Figure 11, and their possible mechanism of action is in Figure 12.

A sulfated polysaccharide (EI-SP) extracted from *Enteromorpha intestinalis* negatively and positively regulated the expression of Bcl-2 and Bax, respectively, generating a loss of mitochondrial membrane potential; therefore, the release of cytochrome *c* to the cytosol, favoring the activation of caspases -9 and -3 of HepG2 cells, culminating the apoptotic process with the cleavage of PARP, chromatin condensation, and nuclear fragmentation, at an IC50 of 98.5 µg/mL [62]. As reported by these authors, EI-SP is a sulfated heteropolysaccharide (16.05% sulfate) composed of 84.76% total sugar (rhamnose, xylose, galactose, glucose, and glucuronic acid), 2.16% protein, and 6.24% uronic acid. There is a relationship between the bioactivity of a polysaccharide and its sulfate content. The higher the sulfate content, the better the biological activity of sulfate [108]. Partial desulfation of sulfated polysaccharides of marine origin was performed. They found that the same polysaccharide with partial desulfurization has significantly lower antioxidant activity than the non-desulfurized polysaccharide, implying that the sulfate content affects the antioxidant activity of the polysaccharide [109]. That result is due to a sulfate group in the polysaccharide that activates hydrogen in the anomeric carbon, enhancing its hydrogen delivery capacity and increasing its antioxidant activity [62]. Second, the higher sulfate content will likely improve the polysaccharide’s water solubility and physicochemical characteristics, thereby increasing its biological activity [110]. In summary, this IC50 below 100 µg/mL is likely due to sulfation of this.

Carrageenans (**Q**) are sulfated polysaccharides present in red algae. They are linear anionic compounds due to the content of 15–40% ester-sulfate, hydrophilic, high molecular weight constituted from alternative units of D-galactopyranose and 3,6-anhydrogalactopyranose, bound to α-1.3 and β-1,4-glucosidic bonds [111]. Sulfated polysaccharides isolated from red algae possess antiproliferative and antitumor characteristics. An extract of sulfated carrageenan (ESC) from *Laurencia papillosa* positively regulated the expression of p53, increased the concentration of ROS, and increased the expression of Bax transcription (4.76), which induces cell death. It decreased the expression of Bcl-2, which resulted in mitochondrial pore formation and, therefore, the release of cytochrome *c*. The apoptotic process was completed by activating caspases -9 and -3 of MDA-MB-231 cells with an IC50 of 50 µg/mL [63] (Figure 12). The authors explained that this imbalance between Bax (pro-apoptotic) and Bcl-2 (anti-apoptotic) might be responsible for the concomitant execution phase of apoptosis, including mitochondrial alterations caused by ROS generation. MDA-MB-231 cells contain mutated and functionally inactive TP53 [112]; therefore, increased expression of this protein after ESC treatment contributes to ESC-dependent apoptosis.

Fucoidan (**R**) is a sulfated polysaccharide in the macroalgae belonging to the *Ochrophyta* division. Its structure and composition depend on the species and biotic and abiotic factors. However, it generally comprises sulfate groups linked to repetitive L-fucose units and different ratios of D-galactose, D-mannose, D-xylose, and uronic acid [111]. Fucoidan possesses anticancer activities, and its bioactivity depends on its constituent monosaccharides [113]. According to [114], the fucose content of polysaccharides affects other physiological activities. Numerous studies have demonstrated the anticancer activity of fucoidan [115]. Overproduction of ROS, loss of mitochondrial membrane potential, negative regulation of c-Myc and Bcl-2 expression, and increased Bax levels were reported using fucoidan with an IC50 of 100 µg/mL on bladder carcinoma 5637 cells [67]. These authors recorded that the maximum generation of ROS in response to fucoidan occurred at 30 min. With an IC50 of 25 µg/mL, fucoidan upregulated the expression of Bax and Bid and decreased the expression of Bcl-2 and Bcl-xL in MDA-MB-231 cells [68]. In addition, it released cytochrome *c*, AIF, and Smac/Diablo to the cytosol and increased the expression of caspases -9 and -3 [68] (Figure 12). Likewise, fucoidan extracted from *Fucus vesiculosus* regulated positively and negatively the expression of Bax and Bcl-2, respectively, of LM3 cells. Moreover, it activated caspases -9 and -3 and reduced the growth of LM3 xenograft tumors in naked athymic mice with an IC50 of 300 µg/mL [69].

Fucoidan reported by Yang et al. consisted mainly of carbohydrates (68.37%), sulfates (21%), protein (0.85%), and uronic acid (10.89%), with fucose and galactose being the main monosaccharides (IC50: 1000 µg/mL) [64] while that reported by *Duan* et al. consisted of 44.1% fucose, 31.1% ash and 26.3% sulfate added with a small amount of amino glucose (IC50: 300 µg/mL) [69]. The fucoidan with the highest percentage of sulfation was 2.5 times more effective than the other. SPs have become a primary research area due to their unique structures and their antioxidant, antitumor, immunomodulating, anti-inflammatory, anticoagulant, antiviral, antiprotozoal, and antibacterial activities [116].

The effect of fucoidan on different tumor cell lines such as prostate adenocarcinoma (DU-145 and PC3), leukemia (SKM-1), hepatocarcinoma (LM3 and SMMC-7721), and mammary adenocarcinoma (MDA-MB-231) has been studied. Fucoidan had a better cytotoxic effect on MDA-MB-231 cells with an IC50 of 25 µg/mL inducing apoptosis by the intrinsic pathway, against an IC50 between 100–1000 µg/mL for the other lines. In SMMC-7721 cells, fucoidan induced apoptosis in both apoptosis-signaling pathways with an IC50 of 560 µg/mL. These results reflect the susceptibility of MDA-MB-231 to treatment and resistance of other cell types, and increased concentration could likely induce apoptosis of these cells through the extrinsic pathway.

Laminarin (**S**) is a storage β-glucan in brown algae. It is a low molecular weight polysaccharide, approximately five kDa, with a variable structure depending on the source. However, its basic form consists of residues of (1,3)-β-D-glucopyranose with branched part 6-O in the primary and β-(1,6)-interfilament bonds [117]. It possesses anti-inflammatory, anticoagulant, antioxidant, and anticancer properties. Different studies have reported its effectiveness against colon carcinoma [70,71] and breast cancer [118]. At an IC50 of 800 µg/mL, laminarin (**S**) increased the concentration of intracellular Ca^2+^ and ROS, activated mitochondrial permeability transition pores, and consequently increased mitochondrial membrane permeability and release of cytochrome *c* to the cytosol and culminated the apoptotic process by activating the -9 and -3 caspases of LoVo cells. ROS percentages were 58.1, 78.6, and 85.1% using 400, 800, and 1600 µg/mL concentrations, respectively [70] (Figure 10). According to the authors, increased ROS can damage mitochondrial membranes, opening mitochondrial permeability transition pores, releasing Ca^2+^ and cytochrome *c*, or increasing Bax expression and producing homodimers acting on such transition pores [70]. Those events decrease the potential of the mitochondrial membrane and, consequently, the release of factors promoting apoptosis. Moreover, increased Ca^2+^ can increase intracellular H+, resulting in the acidification of cells [119]. Therefore, it is likely that laminarin (**S**) has caused acidification in the cell, which on the one hand, could activate DNase II and cause DNA degradation, and on the other hand, facilitate the release of cytochrome c from mitochondria and culminates the automatic execution of caspase apoptosis [70].

At an IC50 800 µg/mL, laminarin (**S**) positively regulated the expression of TRAIL, DR4/5, FADD, and pro-caspase -8, increased the expression of Bid and tBid, increased intracellular Ca^2+^ and ROS, and activated caspase -9 of LoVo cells, and culminated the apoptotic process by activating caspases -3, -6, and -7 [71]. The laminarin extracted from *Laurencia digitata* increased the concentration of Ca^2+^ and caused the loss of the mitochondrial membrane potential of ES2 and OV90 cells using an IC50 of 2000 μg/mL (Figure 10). In addition, laminarin induced apoptosis through ROS generation, endoplasmic reticulum (ER) stress, and DNA fragmentation [72]. The ER is considered a store of Ca^2+^, and an alteration in the level of Ca^2+^ in the ER induces ER stress, which can activate the caspase -12 located in the ER, promoting apoptosis in a way independent of the mitochondria [120,121]. It is essential to underline that intrinsic apoptosis is associated with mitochondrial calcium concentration overload, where such excess can affect the release of pro-apoptotic factors by mitochondrial destruction [72]. These sulfated polysaccharides (SP) mentioned above caused mitochondrial dysfunction with an IC50 of 50 µg/mL, causing an increase in the cytoplasmic calcium concentration in cancer cells leading to apoptosis. Intracellular Ca^2+^ modulates ROS generation and ROS removal processes, thus changing the redox state to a more oxidized or reduced state [122]. Also, sulfated polysaccharides promote ATP synthesis and ROS generation in mitochondria by stimulating Krebs cycle enzymes and oxidative phosphorylation [123] and by regulating multiple extramitochondrial ROS-generating enzymes, including nicotinamide adenine dinucleotide phosphate (NADPH) oxidase (NOX) [124] and nitric oxide synthase (NOS) [125], in physiological and pathological processes. Therefore, an increase in the concentration of Ca^2+^ favors an increase in ROS, leading to the triggering of mitochondria-mediated apoptosis [126].

The anticancer activity of standard laminarin and modified laminarin (modification of sulfate content by chlorosulfonic acid-pyridine method) in LoVo cells was studied by the 3-(4,5-dimethylthiazol-2-yl)-2,5-diphenyltetrazolium (MTT) bromide assay. At 1600 μg/mL, modified laminarin reduced cell viability by 86%, while at the same concentration, unmodified laminarin decreased it by only 38% [127]. These polysaccharides exhibit antioxidant activities closely related to molecular weight and −OSO_3_H content [128]. Different studies have shown that low molecular weight sulfated polysaccharides (SP) have better antioxidant activity than high molecular weight SPs. These SPs from seaweed are essential free radical scavengers [111]. The effect of laminarin on different colorectal carcinoma cell lines (LoVo, ES2, and OV90) has been studied. Laminarin had a better cytotoxic effect on LoVo with an IC50 of 800 µg/mL versus an IC50 of 2000 μg/mL in ES2 and OV90, inducing apoptosis through both pathways only in LoVo. Laminarin in the three different colon cell lines was ineffective as a cytotoxic agent because the IC50 values were above 100 µg/mL. Therefore, studies of this compound are necessary on other tumor cell lines that are probably more susceptible to this compound.

A complex of proteins and selenylated polysaccharides (Se-PPC) extracted from *Ulva fasciata* increased the expression of Fas, activated caspase -8 of the extrinsic pathway, which, in turn, cleaved the pro-apoptotic protein Bid, which amplified the apoptotic response by activating the intrinsic pathway. On the other hand, it increased the expression of p53, which, in turn, decreased the expression of Bcl-2 and Bcl-xL. The overproduction of ROS and the positive regulation of Bax caused by Se-PPC leads to the release of cytochrome *c* to the cytosol, resulting in the activation of caspase -9 of the intrinsic pathway, and, therefore, the activation of caspase -3 generating in such a way the apoptosis of A549 cells to an IC50 of 2.5 μg/mL [73] as morphological changes characteristic of apoptosis, cell contraction and formation of apoptotic bodies occurred. Se-PPC induced depolarization of the mitochondrial membrane of A549 cells. This depolarization was from 6.80% (control) to 16.55, 30.40, and 46.09% at concentrations of 4, 8, and 16 μg/mL, respectively, of Se-PPC. SP) and polysaccharide-protein complex (PPC) exhibit antitumor, immunomodulatory and antioxidant effects [129]. The main polysaccharide of the genus *Ulva* is ulvan (**T**), a sulfated polysaccharide (SP), which accounts for 38–54% of the dry mass. It is composed mainly of L-rhamnose, sulfated rhamnose-3, D-glucose, D-glucuronic acid, and a minor fraction of D-xylose.

Regarding ulvan (**T**), its low molecular weight could increase the degree of sulfation in addition to the uronic acid content, which seems to increase the compound’s antioxidant activity [111]. The antioxidant activity of ulvan (**T**) is due to its ability to eliminate free radicals and metal chelators, in addition to possessing a more significant antioxidant activity in eliminating free radicals than vitamin C, which is conventionally used as an antioxidant [130]. The antioxidant activity of ulvan (**T**) has been reported to cause the antiproliferative effect of sulfated polysaccharides [131,132], and its antitumor activity is a result of sulfation and uronic groups [131] (Figure 12).

On the other hand, selenium (Se) can be highlighted as an essential micronutrient that plays a vital role in different physiological functions in both animals and humans and in preventing and treating cancer [133]. It can form complexes with other molecules, such as polysaccharides and proteins, which are more potent than Se, polysaccharides, or proteins. Selenylated polysaccharides show higher antioxidant, anticancer, immunomodulating, and hepatoprotective bioactivity than native polysaccharides [134,135] due to the synergic activity of Se and polysaccharides [136,137,138]. It is worth mentioning that selenoenzymes and selenopolysaccharides maintain cell membrane integrity and protect lipids, lipoproteins, and DNA from oxidative damage [139]. According to the authors, the Se-PPC of *U. fasciata* contained 44.4 μg/g of Se [73]. The toxicity of Se may be due to the induction of oxidative stress and disruption of redox homeostasis [140], and ROS generation acts as an important cellular event induced by Se, resulting in cell apoptosis and/or cell cycle arrest [141]. Selenopolysaccharides have been shown to exert antitumor activity on breast adenocarcinoma, lung carcinoma, and ovarian cancer [135]. In general, organic selenium is present as selenoamino acids, of which methylselenocysteine, the precursor of methylselenol or methylselenic acid, is necessary for anticancer activity. It has been reported that methylselenol generates an overproduction of reactive oxygen species [142] that probably leads to DNA fragmentation that consequently generates a signal of DNA damage causing the activation of p53 and triggering the entire apoptotic process. Along with the beneficial effects of selenium, the antioxidant potential of ulvan confirms what Negreanu-Pirjol et al. said: ulvan can be used as a potential medicinal compound that could control cancer progression, immunomodulatory and antitumor activity [111].

In summary, the effects of sulfated polysaccharides on different cell lines confirm their protective and repairing effects on cells damaged by oxidative stress and can be used as chemopreventive and/or therapeutic adjuvants in cancer treatment.

### 3.6. Terpenes

**Figure 13:**
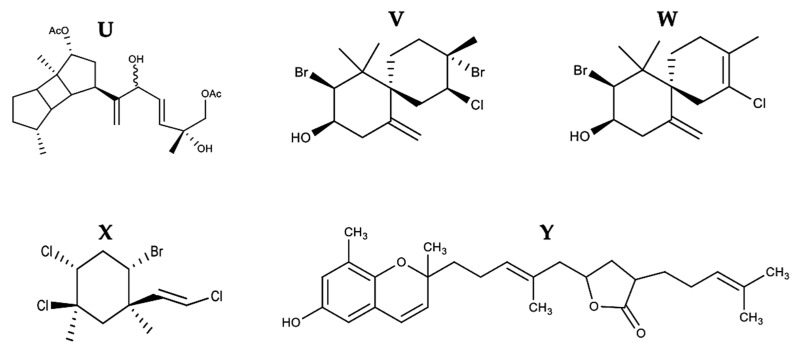
Chemical structure of terpenes extracted from macroalgae with apoptotic activity. (**U**): DDSD; (**V**): Obtusol; (**W**): Elatol; (**X**): Mertensene; (**Y**): Tuberatolide B (TTB).

**Figure 14:**
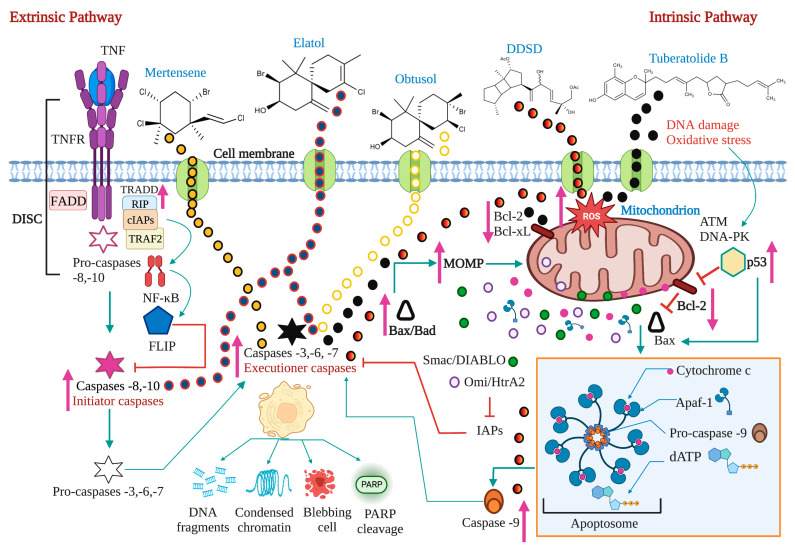
Possible mechanism of action of terpenes extracted from macroalgae inducing apoptosis. It represents the mechanism of action of some compounds such as DDSD, Tuberatolide B, elatol, obtusol, and mertensene in the extrinsic and intrinsic apoptosis pathway, modulating target molecules involved in them. DDSD and Tuberatolide B generate an overproduction of ROS intracellular, which causes mitochondrial dysfunction allowing the release of pro-apoptotic compounds to the cytosol, triggering apoptosis. Pink arrows indicate positive or negative regulation of crucial proteins by such terpenes. Apaf-1: apoptotic protease activating factor-1; ATM: Ataxia-telangiectasia-mutated; Bax: Bcl-2-associated X protein; Bcl-2: B-cell lymphoma; Bcl-xL: B-cell lymphoma-extra-large; Bid: BH3- interacting domain death agonist; Caspase -3, -6, -7, -8, -9, -10: Cysteinyl aspartic acid-protease -3, -6, -7, -8, -9, -10; DNA-PK: DNA-dependent protein kinase; DR 4/5: Death receptor 4/5; FADD: Fas-associated death domain; FLIP: (FADD-like IL-1β-converting enzyme)-inhibitory protein; HtrA2: High-temperature requirement protein A2; IAP: Inhibitors of Apoptosis Proteins; MOMP: Mitochondrial Outer Membrane Permeabilization; NF-κB: nuclear factor kappa-light-chain-enhancer of activated B cells; PARP: poly (ADP-ribose) polymerase; RIP: Receptor interacting protein; SMAC/DIABLO: Second mitochondrial activator of caspases/direct IAP binding protein with low PI; tBid: Truncated Bid.

Terpenoids are widely available phytochemicals in seaweed. They are primarily divided into mono-, di-, tri-, tetra-, grouper-, and sesquiterpenoids. They are excellent antioxidants and exert anticancer activity in vitro and in vivo models [143]. Terpenoids have been shown to inhibit the growth of various cancer cells, such as mammary, skin, lung, colon, pancreas, prostate, and anterior stomach carcinomas, which could be used in cancer therapy [144]. The chemical structure of some terpenes with pro-apoptotic activity is shown in Figure 13 and Figure 14, respectively.

5(R),19-diacetoxy-15,18(R and S), dihydro spata-13, 16(E)-diene (DDSD) (**U**) extracted from *Stoechospermum marginatum*, induced the generation of ROS, decreased and increased the expression of Bcl-2 and Bax, respectively, causing the loss of mitochondrial membrane potential, resulting in the release of cytochrome *c* to the cytosol, and culminated the apoptotic process of B16F10 cells generating the externalization of phosphatidylserine (PS), nuclear condensation, and DNA fragmentation, with an IC50 of 3.7 µg/mL [74] (Figure 14). The effect of two sesquiterpenes, obtusol (**V**) and elatol (**W**), extracted from *Laurencia dendroidea,* was reported on Colo-205 cells [75]. With an IC50 of 1.35 µg/mL, obtusol (**V**) activated caspase -6 and induced 79% apoptosis at the concentration of 100 µg/mL, while elatol (**W**) induced 95% apoptosis at the same concentration and activated caspases -2, -4, -8, and -6 with an IC50 of 2.52 µg/mL [75] (Figure 14). The genus *Laurencia* is one of the largest producers of halogenated substances in the marine environment. Over 5000 halogenated natural products have been identified, and approximately 10% are derived from this genus [145]. The anticancer properties of sesquiterpenoids have been reported to be chemically mediated by the active motifs α-methylene-γ-butyrolactone [146]. Mertensene, extracted from *Pterocladia capillacea*, induced apoptosis with an IC50 of 90 μg/mL on HT-29 cells, upregulating TRADD expression [76] (Figure 14). Mertensene (**X**) is a halogenated monoterpene composed of three chlorine atoms and one bromine atom. Given these characteristics, it is likely that this compound has entered the cell by passive diffusion coupling to TRADD, triggering the apoptotic process. Tuberatolide B (TTB) (**Y**), extracted from *Sargassum macrocarpum*, induced apoptosis in MDA-MB-231, A549, and HTC116 cells at an IC50 of 25.35, 20.28, and 19.86 µg/mL, respectively. ROS generation in MDA-MB-231, A549, and HTC116 cells increased to 67, 36, and 52%, respectively, decreased Bcl-2 expression of the intrinsic pathway, and culminated apoptosis with caspase-3 activation, PARP cleavage, and PS externalization [77] (Figure 14). ROS generation by TTB (**Y**) was the critical modulator for inducing apoptosis. The effect of TTB on different tumor cell lines, such as colon carcinoma (ES2 and OV90), carcinoma of the cervix (HeLa), and leukemia (HL-60), has been studied. TTB had a better cytotoxic effect on HTC116 versus A549 and MDA-MB-231 with an IC50 of 19.86 µg/mL against 20.28 and 25.35 µg/mL, respectively. More studies of TTB are needed because it is an interesting compound for developing anticancer drugs, which registered an IC50 below 26 µg/mL (potent cytotoxicity) on the different tumor lines. These reports confirm that terpenoids are potential chemotherapeutic and/or chemopreventive agents against cancer.

## 4. Discussion and Conclusions

A literature review of the articles published in the last 12 years has been conducted, which has focused on bioactive compounds extracted from macroalgae with the most promising pro-apoptotic effect, emphasizing the regulation of proteins, genes, and key agents in one or both pathways of apoptosis. Twenty-four compounds were of specific interest. The compounds belong to the three main divisions of macroalgae: division *Chlorophyta* (green macroalgae), *Rhodophyta* (red macroalgae), and *Ochrophyta* (brown macroalgae). The pro-apoptotic effect of carotenoids, phenolic compounds, phytosterols, glycoproteins, polysaccharides, and terpenes on different tumor cell lines has been discussed. Colon carcinoma (LoVo, ES2, HT-29, LoVo, OV90, and HCT116), hepatocarcinoma (HepG2, BeL-7402, LM3, and SMMC-7721), and breast adenocarcinoma (MDA-MB-231, MCF-7, and 4T1) were the most reported. The different compound mechanisms of action and structure-activity relationship have also been discussed.

Among the different categories of compounds listed above, polysaccharides are the most studied, with reports on fucoidan and laminarin being the most continuous, followed by a smaller number of carotenoids, phytosterols, and terpenes. The 24 bioactive compounds were divided into five groups according to IC50. The first group contains eight of them who presented an IC50 below 7 µg/mL, from 0.65 to 6.16 µg/mL, being fucoxanthin (**B**) was the only compound reported with an IC50 below 1 µg/mL (IC50 = 0.65 µg/mL) that induced apoptosis to HeLa cells, the second group with 12 compounds which presented an IC50 of 8.7 to 25 µg/mL, the third group with four compounds that showed an IC50 of 32.94 to 50 µg/mL, the fourth group consisting of six compounds, with an IC50 of 55 to 100 µg/mL, and the last one where the remaining compounds and articles were included, being two compounds, fucoidan, and laminarin, with an IC50 of 200 to 2000 µg/mL. Of these compounds, the effect of fucosterol (**K**) and TTB (**Y**) on four and three different tumor cell lines, respectively, presented a potent cytotoxicity with an IC50 below 26 µg/mL. Of the 24 compounds, Se-PPC (complex of proteins and selenylated polysaccharides) was considered the most potent compound because it is the only one with an IC50 of 2.5 µg/mL, which regulated the primary proteins and critical genes of both apoptosis pathways. It activated death receptors and caspase -8 of the extrinsic pathway, increased the expression of p53, which, in turn, decreased the expression of anti-apoptotic proteins such as Bcl-2 and Bcl-xL, and upregulated the expression of pro-apoptotic proteins such as Bax, which was translocated to the mitochondria, leading to pore formation, releasing cytochrome *c* to the cytosol which resulted in the activation of caspase -9 of the intrinsic pathway, and consequently the activation of caspase -3, thus generating apoptosis of A549 cells with cell contraction and formation of apoptotic bodies. It also led to the caspase-8-mediated truncation of the pro-apoptotic protein Bid to amplify the apoptotic response. This effect is due to the Se and the ulvan polysaccharide that make up the complex. Only four compounds of the 24, including Se-PPC, phloroglucinol, fucoidan, and laminarin, could activate Bid truncation via caspase -8 and thus amplify the mitochondrial pathway.

It is important to mention that all categories of compounds induced apoptosis through the intrinsic mitochondrial pathway, regulating the pro- and anti-apoptotic proteins of the Bcl-2 family, culminating this process by activating effector caspases (-3, -6, and -7). On the other hand, polysaccharides were better able to regulate intracellular ROS and Ca^2+^ levels than the other groups. Polysaccharides, phytosterols, and glycoproteins were more effective in causing the mitochondria’s dysfunction and releasing apoptotic compounds to the cytosol by causing the loss of mitochondrial membrane potential. In contrast, carotenoids, phenolic compounds, and polysaccharides regulated the death receptors of the extrinsic pathway better than the other groups.

Regarding cancer cell lines, four studies were recorded on MDA-MB-231 breast cancer cells. The terpene TTB and ESC demonstrated better efficacy than fucoidan and GLP (glycoprotein) with an IC50 of 25 µg/mL against 50 and 55 µg/mL, respectively, with apoptosis induced by the intrinsic pathway in all cases. This result may be associated with the susceptibility of MDA-MB-231 cells to TTB and ESC. On the other hand, studies on TTB, ESC, and GLP of macroalgae with anticancer properties are minimal, so they should be a new object of study. Regarding HepG2 hepatocarcinoma, β-carotene had a better cytotoxic effect than HFGP (glycoprotein) and EI-SP (sulfated polysaccharide), with an IC50 of 5.63 against 25 and 98.4 µg/mL, respectively, registering apoptosis by both routes only by the effect of β-carotene and HFGP. As for A549 lung cancer, three studies were recorded. Se-PPC was more effective than TTB and Dieckol because it presented an inhibitory concentration of 2.5 µg/mL against 20.28 and 25 µg/mL, respectively; Se-PPC was the only one that induced apoptosis on A549 cells through both routes. Each cell line has its characteristics and responds differently to treatments. A compound may have a good effect on one and not the other cell death pathway. Future research must identify specific compounds or combinations corresponding to each cell line’s molecular setup.

One of the main characteristics that stood out from the different compounds is their antioxidant and pro-oxidant capacity. Overproduction of ROS causes oxidative stress, which is involved in the development of various diseases, mainly cancer, so antioxidants are appreciated not only for their ability to eliminate free radicals but also for their participation in numerous functions in signal transduction pathways. Of the 24 compounds reported, 11 of them acted as pro-oxidants increasing intracellular ROS levels. Because of oxidative stress, pores along the mitochondrial membrane were oxidized or depolarized the mitochondrial membrane and thus activated the intrinsic pathway of apoptosis by causing mitochondrial dysfunction, which led to the release of pro-apoptotic compounds from the mitochondria to the cytosol. Dieckol (phlorotannin), fucosterol (phytosterol), GLP (glycoprotein), TTB (terpene), DDSD (terpene), fucoidan (polysaccharide), Se-PPC (polysaccharide with proteins and Se), laminarin (polysaccharide) and ESC (polysaccharide) can be cited as compounds with pro-oxidant activity.

On the other hand, the structure-activity relationship of different compounds was highlighted, where the antioxidant activity of a compound is closely linked to its anticancer activity because eliminating oxidants is essential to control cancer. The antioxidant capacity of phenolic compounds is due to the presence of their hydroxyl group, their highly hydrophilic components, and their site of action. In contrast, the antioxidant capacity of polysaccharides is highly related to their degree of sulfation, relative molecular mass, dominant sugar type, and glycosidic branching. The study of sulfated polysaccharides continues to increase because sulfation improves biological activity. The higher the sulfate content, the better the activity.

It is important to note that most of the promising compounds, such as fucosterol, fucoxanthin, fucoxanthinol, siphonaxanthin, TTB, DDSD, dieckol, mertensene, ulvan, fucoidan, and laminarin, described in this research do not belong to classes of metabolites that are biosynthesized in terrestrial species, the most evident being halogenated terpenes and polysaccharides. This fact reinforces the importance of the marine environment, and macroalgae, as a source of potential new cancer drugs. However, more in-depth investigations of these compounds are necessary to understand the activation and modulation of the target molecules of both apoptosis signaling pathways, the structure-activity relationship, and the evaluation of the cytotoxicity of these compounds in non-tumor cells. Likewise, evaluating the most promising compounds on other tumor cell lines and in vivo studies, the synergy of these with first-line drugs, and long-term drug delivery are necessary because some of these compounds exhibit activities that warrant greater attention from researchers and the pharmaceutical industry.

## Data Availability

Data sharing is not applicable.
